# The Relationships of Leaders' Narcissistic Admiration and Rivalry with Nurses' Organizational Citizenship Behavior towards Leaders: A Cross-Sectional Survey

**DOI:** 10.1155/2023/5263017

**Published:** 2023-08-30

**Authors:** Yanghao Zhu, Yannan Zhang, Feng Qin, Yang Li

**Affiliations:** ^1^School of Management, Huazhong University of Science and Technology, Wuhan, Hubei, China; ^2^School of Business Administration, Southwestern University of Finance and Economics, Chengdu, Sichuan, China; ^3^School of Management Jiujiang University, Jiujiang, Jiangxi, China; ^4^School of Business Administration, Zhongnan University of Economics and Law, Wuhan, Hubei, China

## Abstract

**Aim:**

The aim of this study is to investigate the unique contributions of two distinct dimensions of leaders' narcissism—admiration and rivalry—to nurses' organizational citizenship behavior towards the leader and the mediating role of nurses' challenge appraisal and hindrance appraisal based on the transactional stress theory.

**Background:**

Leaders' narcissism is widespread in hospitals, so whether nurses will show organizational citizenship behavior to narcissistic leaders is related to the harmony and stability of the team. Thus, clarifying the positive or negative relationship between leaders' narcissism and nurses' organizational citizenship behavior towards leaders has become a priority.

**Methods:**

A cross-sectional survey was conducted at two-time points with 280 Chinese nurses. We used the structural equation model to analyze our data.

**Results:**

We found that leaders' narcissistic admiration was positively correlated with nurses' organizational citizenship behavior towards the leader; however, leaders' narcissistic rivalry was negatively correlated with nurses' organizational citizenship behavior towards the leader. Furthermore, mediation analyses revealed that leaders' narcissistic admiration had a significant indirect effect on nurses' organizational citizenship behavior towards leaders via challenge appraisal, and leaders' narcissistic rivalry had a significant indirect effect on nurses' organizational citizenship behavior towards leaders via hindrance appraisal.

**Conclusion:**

By introducing two dimensions of narcissistic admiration and rivalry, we resolve previous contradictory findings about the relationship between leader narcissism and organizational citizenship behavior towards the leader. *Implications for Nursing Management*. Nursing managers need to understand that there are both positive and negative relationships between leaders' narcissism and nurses' organizational citizenship behavior towards the leader, and we can promote the positive effects of leaders' narcissistic admiration and curb the negative consequences of leaders' narcissistic rivalry by taking some specific steps, such as promoting the loving cooperative atmosphere and punishing disruptive behaviors.

## 1. Introduction

Narcissism can be characterized by an inflated self-view, a strong sense of psychological superiority and entitlement, and a low level of empathy [1, 2]. In hospitals, narcissism is quite prevalent [3], especially for leaders [4]. Therefore, it is inevitable that nurses have interpersonal interactions with narcissistic leaders. Because narcissistic leaders are self-centered, they expect nurses to perform more extra-role behaviors [5], in particular organizational citizenship behavior toward leaders (OCBL), to meet their own unique needs. Only by understanding the consequences of narcissistic leaders in the process of interpersonal interaction (e.g., nurses' OCBL), can the organization give play to the positive effects of narcissistic leaders and restrain its negative consequences, so as to improve the harmonious atmosphere and cohesion of the team.

However, we found by reviewing previous studies that the relationship between leader narcissism and OCBL seems to have contradictory findings. On the one hand, narcissistic leaders focus on themselves, neglect the interests and feelings of others, and lack empathy, leading to a decrease in OCBL. For example, some scholars confirmed that leader narcissism negatively affects OCBL by increasing hindrance stress [6] and decreasing perceived insider status [7]. On the other hand, some attributes of narcissistic leaders, such as self-confidence, boldness of vision, and a strong desire for leadership and success, produced productive or positive outcomes [8]. Some scholars found that narcissistic leaders are positively related to change-oriented OCB and questioned the idea that leader narcissism is always negatively associated with OCBL [9].

The reason for these contradictory findings can be traced back to the fact that prior research studies conducted on narcissism as a global construct with two facets of narcissism have been neglected [10, 11]. Therefore, we draw on the narcissistic admiration and rivalry concept [12] in which the NARC proposes that narcissism contains two related dimensions: an agentic dimension called narcissistic admiration and an antagonistic dimension called narcissistic rivalry ([13], p. 59). Narcissistic admiration involves anticipation and an approach to opportunities for admiration, through assertive self-promotion [12, 14]. Those positive strategies elicit challenge stress assessments from nurses, which in turn leads to positive interpersonal outcomes (e.g., increased OCBL). Conversely, narcissistic rivalry involves striving for supremacy and devaluation of others, through antagonistic self-protection [12, 14]. Those negative strategies elicit hindrance stress assessments from nurses, which in turn leads to negative interpersonal outcomes (e.g., decrease OCBL). Thus, the present study will explain the inconsistencies between narcissistic leaders and nurses' OCBL using the NARC framework.

The paper is organized as follows. In Section 2, the hypotheses are developed. Section 3 includes samples, procedures, and measurement scales. In Section 4 to Section 6, the results are displayed and subsequently discussed in terms of main findings, contributions, and limitations. Finally, the implications for nursing management are provided in Section 7.

## 2. Theory and Hypotheses

### 2.1. Leader Narcissism and Nurses' OCBL

NARC is a bidimensional (i.e., admiration and rivalry) process model of narcissism describing motivational and behavioral dynamics alongside social interaction outcomes [12]. Narcissistic admiration might lead to favorable social outcomes, and the narcissistic rivalry is presumed to go along with exhibiting devaluing behavior toward others and negative social outcomes [12]. Thus, we predicted that leaders' narcissistic admiration positively impacted nurses' OCBL; however, leaders' narcissistic rivalry negatively impacted nurses' OCBL.

Specifically, leaders' narcissistic admiration would be adopting assertive self-promotion strategies, which can be summarized with slogans such as “show the world how great you are!” or “let others admire you!” ([13], p. 59). They strove for uniqueness, actualized grandiose fantasies, and exhibit a charming (expressive, self-assured, and dominant) behavior [12]. In this case, nurses perceive their leader as confident, competent, and charismatic, and these positive characteristics are in line with the leadership prototype expected by nurses, which deepens nurses' favorable impression of their leader and further promotes nurses to implement OCB towards their leaders [15]. However, leaders' narcissistic rivalry adopts antagonistic self-protection strategies, which can be summarized with the imperative “do not let others tear you down!” ([13], p. 60). They strove for supremacy, devaluate others, and exhibit an aggressive (annoyed, hostile, and socially insensitive) behavior [12]. In this case, nurses perceive their leaders as cold-blooded, hostile, or unsympathetic, and these negative characteristics are inconsistent with the leadership prototype expected by nurses, which deepens nurses' antipathy toward the leader and further decreases nurses' desire to implement OCB towards their leader.

  H1a: leaders' narcissistic admiration positively impacted nurses' OCB towards leaders
  H1b: leaders' narcissistic rivalry negatively impacted nurses' OCB towards leaders

### 2.2. The Mediating Role of Nurses' Challenge and Hindrance Appraisal

To further explore the specific process of leaders' narcissistic admiration and rivalry with nurses' OCBL, this paper constructed a theoretical model of challenge and hindrance appraisal as mediating variables based on the transactional stress theory [16]. The transactional stress theory argues that environmental conditions (termed stressors) are not the direct cause of a stress reaction, but rather it is the people's appraisal of a challenge or hindrance that proceeds the response [17]. LePine et al. [18] pointed out that challenge stressors likely elicit challenge appraisals and hindrance stressors likely elicit hindrance appraisal, and the two appraisals differentially impact individual behaviors. Thus, we predicted that leaders' narcissistic admiration (challenge stressor) positively impacts nurses' OCBL via challenge appraisal and leaders' narcissistic rivalry (hindrance stressor) negatively impacts nurses' OCBL via hindrance appraisal.

Specifically, challenge stressors refer to job demands or environmental situations that require effort but have the potential to create opportunities for performance and support goal pursuits [19, 20]. According to this definition, leaders' narcissistic admiration can be considered a challenging stressor. This is because narcissistic admiration leaders treat their nurses better, strive to maintain interpersonal relationships with nurses, and even take the initiative to help nurses [21]. In this kind of an environmental situation, leaders' knowledge, skills, and abilities can help them achieve personal gain, growth, development, and well-being [18], which generates challenge appraisal. Furthermore, challenge appraisal may induce nurses' positive emotions and intrinsic motivation, so they engage in positive behaviors, and especially when they are influenced by leaders, they are more willing to engage in OCBL. Parker et al. [22] put forward a point that challenge appraisal positively impacts intrinsic motivation and prosocial behavior.

  H2a: challenge appraisal played a mediating role between leaders' narcissistic admiration and nurses' OCB towards leaders

Hindrance stressors refer to job demands or environmental situations that involve excessive or undesirable constraints that interfere with or hinder an individual's ability to achieve valued goals ([19], p. 67). Leaders' narcissistic rivalry can be considered a hindrance stressor because leaders high in narcissistic rivalry are focused on their advantages but lack empathy and concern for nurses, and they build success at the expense of nurses [12, 23]. In this kind of an environmental situation, leaders' narcissistic rivalry threatened nurses' well-being by hindering the attainment of goals and personal development, and nurses generated hindrance appraisals. In addition, hindrance appraisal may induce nurses' negative emotions and they may choose to deal with work in negative ways, including reducing work effort and helping others (i.e., OCBL) [18, 22].

  H2b: hindrance appraisal played a mediating role between leaders' narcissistic rivalry and nurses' OCB towards leaders

**Figure 1:**
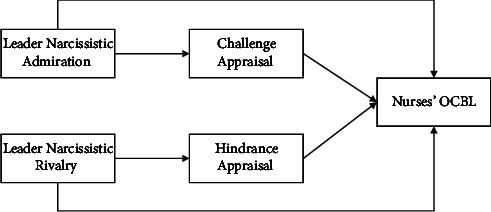
Conceptual framework.

The specific conceptual framework is shown in Figure 1.

## 3. Method

### 3.1. Samples and Procedures

We recruited 350 full-time Chinese nurses to complete the two-stage questionnaires via https://www.credamo.com/, a reliable Chinese data collection platform similar to the Qualtrics online sample. Other studies have used this platform to collect data (i.e., [24, 25]). Only nurses who have stable full-time jobs involving frequent face-to-face communication with their leaders can participate in the follow-up survey as participants. In this study, we adopted the following ways to ensure ethical issues. First of all, nurses were invited to participate in the survey through voluntary registration, and they had the right to quit at any stage of the survey. Second, the survey ensured the privacy of the participants, and all procedures performed involving human participants followed the ethical standards of the institutional and/or national research committee. Finally, at the end of the survey, we informed the participants of our research purpose and context, which was to eliminate the adverse effects caused by participation in the survey.

In the first stage, we collected the demographic variables (age, educational background, and tenure in the organization) of all nurses, and nurses were asked to rate their leaders' narcissistic admiration and rivalry. In the second stage, we asked nurses to provide ratings for challenge appraisal, hindrance appraisal, and OCBL. We eliminated the following three types of invalid questionnaires. First, the questionnaire which was incomplete and more than half of the items in a single scale were not answered. Second, the questionnaires in which the participants failed the attention check. Third, the unmatched questionnaire, that is, where the first stage and the second stage cannot be matched. Finally, we obtained 280 complete and usable questionnaires with a response rate of 80%. As suggested by the reviewer, a post hoc analysis was conducted using Gpower3.1 to determine if the sample size was appropriate. The results showed power = 0.99, which is greater than the standard of 0.80, indicating that the sample size was appropriate. The specific demographic variables are shown in Table 1.

### 3.2. Measure

As the original scales of our study were developed in English, we followed Brislin's (1986) translation and back-translation procedure to ensure the accuracy of translating English items into Chinese. Responses were collected using a seven-point Likert scale ranging from 1 for “strongly disagree” to 7 for “strongly agree.” All scales are unidimensional and have no reverse items.

#### 3.2.1. NARC

We measured NARC by using the eighteen-item scale developed by Back et al. [12]. Nine items assessed narcissistic admiration and nine assessed narcissistic rivalry. According to Fehn and Schütz's [23] conclusion, we adopted the nurse-rated leaders' narcissistic admiration and rivalry. Sample items included the following: “my leader wants his/her rivals to fail (rivalry)” and “my leader shows others how special he/she is (admiration).” Cronbach's *α* = 0.87 (0.80) for rivalry, and Cronbach's *α* = 0.89 (0.84) for admiration in our study (original study).

#### 3.2.2. Challenge and Hindrance Appraisal

We used two three-item scales developed by LePine et al. [18] for challenge and hindrance appraisal. Sample items included the following: “in general, I feel that my job promotes my personal accomplishment (challenge appraisal)” and “in general, I feel that my job hinders my personal accomplishments (hindrance appraisal).” Cronbach's *α* = 0.75 (0.83) for challenge appraisal, and Cronbach's *α* = 0.79 (0.70) for hindrance appraisal in our study (original study).

#### 3.2.3. OCBL

We measured OCBL by using the six-item scale developed by Dalal et al. [26]. Sample items included the following: “I went out of my way to be nice to my leader” and “I tried to help my leader.” Cronbach's *α* = 0.84 (0.83) for OCBL in our study (original study).

#### 3.2.4. Control Variables

Nurse's age, educational background, and organizational tenure were included as control variables in the analyses since they could be related to OCB in prior studies (see, e.g., [27–29]).

## 4. Results

### 4.1. Confirmatory Factor Analysis

We conducted a series of confirmatory factor analyses to assess the discriminant validity of the variables in our model. Due to many measurement items of some variables (i.e., narcissistic admiration and rivalry), considering that there are many parameters to be estimated in the model, the standard errors may be increased. According to Little et al.'s [30] recommendation, narcissistic admiration and rivalry are randomly packaged into three parcels. The fit statistics of a model that included the focal variables (i.e., leaders' narcissistic admiration, leaders' narcissistic rivalry, challenge appraisal, hindrance appraisal, and OCBL) were acceptable: *χ*^2^ (125) = 219.35, RMSEA = 0.05, CFI = 0.95, TLI = 0.94, and SRMR = 0.05. Alternative model specifications resulted in a deteriorated fit statistics. For example, a model combining both the independent variables (narcissistic admiration and rivalry) resulted in a worse-fitting model: *χ*^2^ (129) = 442.55, RMSEA = 0.09, CFI = 0.84, TLI = 0.81, and SRMR = 0.10, as did a model combining both the mediators (challenge appraisal and hindrance appraisal): *χ*^2^ (129) = 377.69, RMSEA = 0.08, CFI = 0.87, TLI = 0.85, and SRMR = 0.07.

### 4.2. Common Method Bias Test

Since all variables in this study were evaluated with nurses, a test for the possible common method bias was required. First, the results of Harman's one-factor analysis indicated that the cumulative variance interpretation rate of the first precipitation factor was 25.14%, which was less than half of the total explanation (62.18%), and did not exceed the recommendation criterion of 40% [31], provisionally indicating that the sample did not have a serious problem of common method bias. Second, by adding a common method factor into the five-factor model, the results show that the six-factor model could not be fitted by the Mplus8.3 software, again indicating that the sample does not have a serious common method bias problem and can proceed to the next step of data analysis.

### 4.3. Descriptive Statistics

Table 2 provides the means, standard deviations, alphas, and correlations for the study variables. As shown in Table 2, leaders' narcissistic admiration was positively related to challenge stressors (*r* = 0.40 and *p*  < 0.01) and OCBL (*r* = 0.30 and *p*  < 0.01). Leaders' narcissistic rivalry was positively related to hindrance stressors (*r* = 0.26 and *p*  < 0.01) and negatively related to OCBL (*r* = −0.33 and *p*  < 0.01). In addition, challenge appraisal was positively related to OCBL (*r* = 0.52 and *p*  < 0.01); however, hindrance appraisal was negatively related to OCBL (*r* = −0.39 and *p*  < 0.01). These results preliminarily supported our hypothesis.

### 4.4. Hypotheses Testing

We tested the proposed model using structural equation modeling (SEM) with Mplus8.3. Following Anderson and Gerbing [32], we compared a sequence of nested models to identify the best model for testing the proposed hypotheses.

We first tested the direct effect of leaders' narcissistic admiration and rivalry on OCBL in the absence of mediating variables. We found that leaders' narcissistic admiration had a significant positive impact on OCBL (*b* = 0.30, SE = 0.08, and *p*  < 0.01) and leaders' narcissistic rivalry had a significant negative impact on OBCL (*b* = −0.43, SE = 0.09, and *p*  < 0.01), after controlling for nurse age, educational background, and tenure. Hypotheses 1a and 1b were supported. We thus proceeded to investigate whether the relationship was mediated by challenge appraisal and hindrance appraisal.

To determine the optimum structural model fit, fully mediated and partially mediated models were examined. We used the partial mediation model (M0) as the baseline model. In the baseline model, we estimated all relationships between the current variables. In M1 (full mediation model), we removed the direct path from leaders' narcissistic admiration and rivalry to OCBL. Control variables were included in all structural models.  Table 3 shows the results of confirmatory factor analyses (CFAs) of the nested models. The results in Table 3 show that both M0 (*χ*^2^ (178) = 316.62, CFI = 0.93, TLI = 0.91, and RMSEA = 0.05) and M1 (*χ*^2^ (180) = 323.44, CFI = 0.93, TLI = 0.91, and RMSEA = 0.05) fit the data well. Both the fixed indices and the chi-square test (*p*  < 0.05) indicated significant differences between M1 and M0; thus, M0 (partial mediation model) was selected.

**Figure 2:**
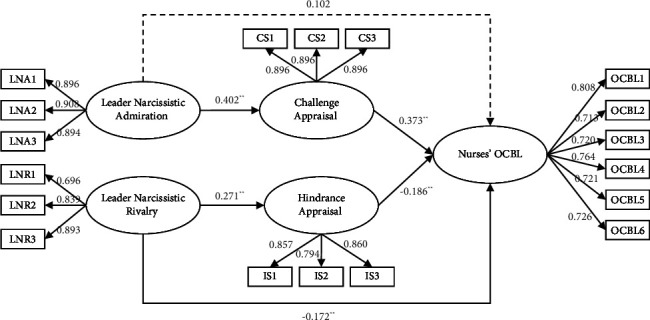
The results of SEM.

We tested this indirect effect of M0 using the procedure developed by Edwards and Lambert [33]. Thus, we calculated the indirect effect of leaders' narcissistic admiration and rivalry on OCBL mediated through challenge appraisal and hindrance appraisal and developed CI with 5,000 resamples. The SEM results are shown in Figure 2. Here, the indirect effect of leaders' narcissistic admiration on OCBL through challenge appraisal was significant (indirect effect = 0.15, 95% CI: 0.094 and 0.213), supporting Hypothesis 2a. Moreover, the indirect effect of leaders' narcissistic rivalry on OCBL through hindrance appraisal was significant (indirect effect = −0.05, 95% CI: −0.119 and −0.011), supporting Hypothesis 2b.

## 5. General Discussion

We have learned about the important role of nurses' OCBL in enhancing and improving the relationship between leaders and nurses, and that leaders' narcissism is one of the important antecedents of nurses' OCBL. Despite the strong interest of theoretical scholars and management practitioners in leaders' narcissism, the existing literature has largely taken a single view of this trait and presented inconsistent findings on its interpersonal impact. In contrast, our study adopts a two-dimensional view of narcissism and outlines how leaders' narcissism positively and negatively impacts nurses' OCBL. Using a multistage questionnaire study of 280 nurses from China, we found that leaders' narcissistic admiration was positively associated with nurses' OCBL, whereas leaders' narcissistic rivalry was negatively associated with nurses' OCBL. In addition, challenge appraisal substantially mediated the relationship between leaders' narcissistic admiration and nurses' OCBL, and hindrance appraisal substantially mediated the relationship between leaders' narcissistic rivalry and nurses' OCBL. This result has great significance.

First, many scholars have explored the relationship between narcissistic leaders and OCBL but have drawn inconsistent conclusions. As demonstrated by our decision to explore leader narcissism and its many implications on nurses' OCBL based on the two-dimensional view of narcissism, we urge scholars to use the method that extensively analyze narcissism [34]. Our study based on Back et al. [12] proposed that narcissism is a two-dimensional concept with both positive and negative traits, divided narcissism into two dimensions of narcissistic admiration and rivalry, and explored the relationship between narcissistic leaders and OCBL. Our results acknowledge and emphasize the need of elaborating narcissists' subtle and fundamental inner conscious patterns in order to better comprehend their range of interpersonal and behavioral tendencies toward other individuals in the workplace [35].

Second, our research adds to the body of knowledge on leadership, notably on leaders' narcissism and the relationship between leaders and nurses. The mechanisms connecting leaders' narcissism and nurses' OCBL are specifically revealed by our study. Furthermore, we contend that this study puts the spotlight on unique and previously unrecognized pathways connected to narcissism in the workplace. For example, previous narcissism-related research has focused on intimate and acquaintance-based relationship networks [14, 36, 37]. These intimate relationships are distinct from work relationships, which are often more distant. Based on the transactional stress theory, our study found that challenge appraisal substantially mediates the relationship between leaders' narcissistic admiration and nurses' OCBL, and hindrance appraisal substantially mediates the relationship between leaders' narcissistic rivalry and nurses' OCBL. In conclusion, we believe that by revealing the unique mechanisms between leaders' narcissism and nurses' OCBL, we have made a contribution to how leaders' narcissism affects interpersonal consequences and advanced the study of narcissism in the field of leadership.

Finally, we have also contributed to the study of the antecedents of OCBL in the field of nursing management. In hospitals, a good or bad relationship between leaders and nurses can affect the working atmosphere of the team or organization and even the functioning of the hospital. Therefore, it is especially important to understand the positive or negative antecedents of nurses' OCBL. On the other side of the leader-nurse interaction, leaders' traits may have a strong impact on nurses' OCBL. Therefore, this study specifically examined the relationship between leaders' narcissistic traits and nurses' OCBL and found that the positive trait of leaders' narcissistic admiration promotes nurses' OCBL, whereas the negative trait of leaders' narcissistic rivalry inhibits nurses' OCBL. In conclusion, our study provides two very important antecedents of OCBL in the field of nursing management.

## 6. Limitations

There are some limitations to consider when evaluating our findings. First, as the data were self-reported, there may be concerns about the common method bias [31]. Although our study confirms that there is no serious common method bias problem, we recommend that future studies use experimental or multistage and multisource longitudinal research designs to enhance the causality of our research conclusions.

Second, according to the conclusion of Fehn and Schütz [23], we use other-rated (nurses) but not self-rated leaders' narcissistic admiration and rivalry. However, we suggest that future studies can continue to compare the impact of self-rated and nurse-rated leaders' narcissistic admiration and rivalry on OCBL and observe whether there is a difference, which is still an interesting topic.

## 7. Implications for Nursing Management

Our study concluded that leaders' narcissistic admiration can increase nurses' OCBL via increasing their challenge appraisal, and leaders' narcissistic rivalry can decrease nurses' OCBL via increasing their hindrance appraisal. In order to improve the quality of leader-member interaction, hospitals should maximize the positive effects of leaders' narcissistic admiration and avoid the negative effects of leaders' narcissistic rivalry.

(1) *For Hospitals.* Hospitals can suppress the expression of the negative effects of narcissistic leaders by implementing both “soft culture” and “hard policy.” Specifically, the hospital should actively promote the organizational culture of cooperation and punish the sabotage and create a working atmosphere of mutual help, solidarity, and love, so as to strengthen the positive relationship between the leaders' narcissistic admiration and nurses' OCBL and weaken the negative relationship between the leaders' narcissistic rivalry and nurses' OCBL.

(2) *For Leaders.* Leaders should lead by example, actively play the positive role of leaders' narcissistic admiration, and try to avoid the negative consequences of leaders' narcissistic rivalry. Specifically, leaders should feel free to display their own charismatic (an external form of narcissistic admiration) and positive narcissistic traits when they want to during their regular interactions with nurses but should refrain from doing so when they want to disparage others. This can help build a more positive leader-nurse relationship.

(3) *For Nurses.* Nurses think that their leader is narcissistic, and there are specific impression management strategies which they can use to deal with it. For instance, nurses may need to emphasize their similar work experiences and backgrounds while speaking with leaders who are narcissistic admiration. Nevertheless, nurses may need to display a little more humility and avoid some behaviors (e.g., self-promotion) while speaking with leaders who are narcissistic rivalry because these leaders may view these behaviors as threatening.

## Figures and Tables

**Table 1:** Demographic characteristics (*n* = 280).

Characteristics	Frequency	%
*Age (years)*		
20–29	118	42.1
30–39	141	50.4
40–49	14	5.0
50–59	7	2.5

*Educational background*		
High school and below	8	2.9
Associate degree	33	11.8
Bachelor's degree	197	70.4
Master's degree and higher	42	15.0

*Tenure (years)*		
1–5	114	40.7
6–10	122	43.6
11–15	28	10.0
>15	16	5.7

**Table 2:** Descriptive statistics, correlations, and reliabilities.

Variables	M	SD	1	2	3	4	5	6	7
1. Age	31.09	6.40							
2. Education	2.98	0.62	−0.09						
3. Tenure	7.29	5.43	0.86^*∗∗*^	−0.16^*∗∗*^					
4. Leaders' narcissistic admiration	5.00	0.95	0.04	0.02	0.05				
5. Leaders' narcissistic rivalry	2.75	0.98	−0.09	−0.04	−0.05	−0.13^*∗*^			
6. Challenge appraisal	5.94	0.46	0.01	0.02	0.01	0.40^*∗∗*^	−0.25^*∗∗*^		
7. Hindrance appraisal	2.35	0.63	−0.06	0.08	−0.02	−0.17^*∗∗*^	0.26^*∗∗*^	−0.39^*∗∗*^	
8. OCBL	5.48	0.67	0.04	0.05	0.03	0.30^*∗∗*^	−0.33^*∗∗*^	0.52^*∗∗*^	−0.39^*∗∗*^

^*∗*^ *p* < 0.05; ^*∗∗*^*p* < 0.01.

**Table 3:** Model comparison results.

Model	*χ* ^2^	*df*	*χ* ^2^ * /df*	CFI	TLI	RMSEA	∆*χ*^2^	∆*df*	AIC
M0	316.62	178	1.78	0.93	0.91	0.05	—	—	15206.23
M1	323.44	180	1.80	0.93	0.91	0.05	2	6.82^*∗*^	15209.05

## Data Availability

The data that support the findings of this study are available from the corresponding author upon request.
